# Cardiometabolic Effects of Postnatal High-Fat Diet Consumption in Offspring Exposed to Maternal Protein Restriction *In Utero*

**DOI:** 10.3389/fphys.2022.829920

**Published:** 2022-05-10

**Authors:** Aiany Cibelle Simões-Alves, Ana Paula Fonseca Cabral Arcoverde-Mello, Jéssica de Oliveira Campos, Almir Gonçalves Wanderley, Carol Virginia Gois Leandro, João Henrique da Costa-Silva, Viviane de Oliveira Nogueira Souza

**Affiliations:** ^1^ Laboratory of Nutrition, Physical Activity and Phenotypic Plasticity, Department of Physical Education and Sport Sciences, Universidade Federal de Pernambuco UFPE, Vitória de Santo Antão, Brazil; ^2^ Department of Physiology and Pharmacology, Universidade Federal de Pernambuco UFPE, Recife, Brazil

**Keywords:** hypertension, obesity, nutrition transition, saturated fatty acids, dyslipidemia

## Abstract

In recent decades, the high incidence of infectious and parasitic diseases has been replaced by a high prevalence of chronic and degenerative diseases. Concomitantly, there have been profound changes in the behavior and eating habits of families around the world, characterizing a “nutritional transition” phenomenon, which refers to a shift in diet in response to modernization, urbanization, or economic development from undernutrition to the excessive consumption of hypercaloric and ultra-processed foods. Protein malnutrition that was a health problem in the first half of the 20th century has now been replaced by high-fat diets, especially diets high in saturated fat, predisposing consumers to overweight and obesity. This panorama points us to the alarming coexistence of both malnutrition and obesity in the same population. In this way, individuals whose mothers were undernourished early in pregnancy and then exposed to postnatal hyperlipidic nutrition have increased risk factors for developing metabolic dysfunction and cardiovascular diseases in adulthood. Thus, our major aim was to review the cardiometabolic effects resulting from postnatal hyperlipidic diets in protein-restricted subjects, as well as to examine the epigenetic repercussions occasioned by the nutritional transition.

## Introduction

**FIGURE 1:**
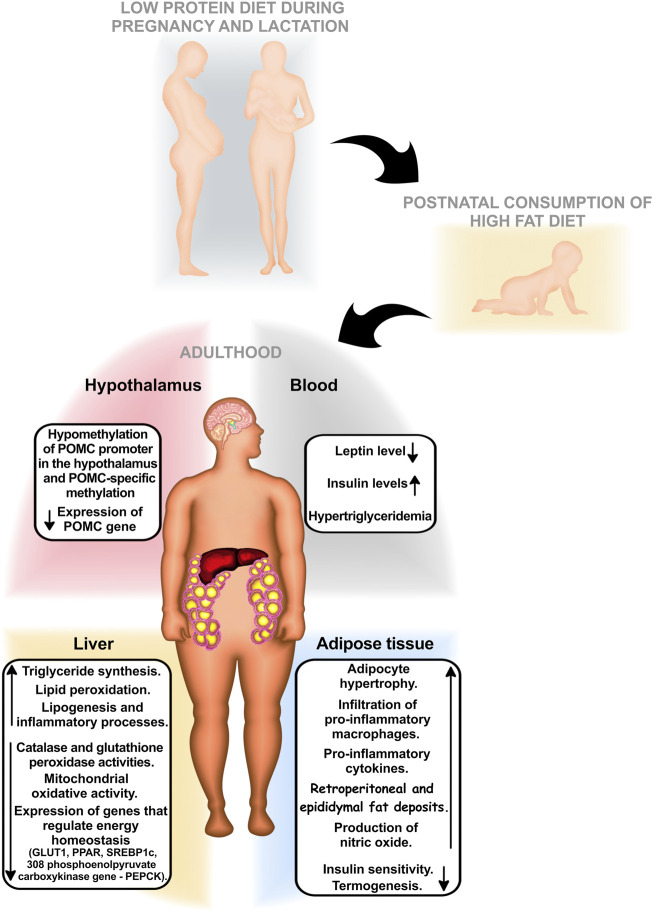
Diets low in protein in the first years of life, followed by high consumption of high-fat diets, can increase the risk of cardiometabolic diseases in adulthood, such as hypertension, dyslipidemia, and type 2 diabetes.

In the second half of the 20th century, a change in the dietary habits of the population, mainly in the western part of the world, was observed, called a phenomenon of “nutrition transition” (Hasan et al., 2016; Herran et al., 2016). The nutritional transition was characterized by a reduction in the prevalence of malnutrition in its various dimensions (energy and macro- or micronutrients), accompanied by excessive consumption of hypercaloric and ultra-processed foods, with a consequent increase in body weight (Abdullah, 2015), a high incidence of chronic diseases, and a high prevalence of obesity (Batista Filho and Rissin, 2003a; Batista Filho and Batista, 2010; Ribeiro et al., 2015). Concomitantly, it was possible to observe profound changes in the behavior and eating habits of families around the world (Popkin, 2015). Higher levels of undernutrition have been replaced by higher rates of overweight and obesity related to hyperlipidic and hypercaloric food consumption (Di Pietro et al., 2015). Epidemiological evidence has shown that nutritional deficiency in the first years of life accompanied by overnutrition, a *posteriori*, may increase the risk of dyslipidemia and other cardiometabolic diseases in adulthood, such as hypertension and type 2 diabetes (Figure 1) (Barker, 2007). In this context, individuals subjected to maternal protein undernutrition *in utero* have been considered those with a high risk of developing cardiometabolic dysfunctions in adulthood (Ashton, 2000; Hemachandra et al., 2006; Antony and Laxmaiah, 2008; Conde and Monteiro, 2014; Costa-Silva et al., 2015; Parra et al., 2015). Furthermore, studies have suggested that when these individuals are additionally subjected to inappropriate postnatal nutrition, especially hyperlipidic diets, they may significantly suffer heightened energy balance dysfunctions in adulthood (Desai et al., 2007). Thus, the eating habits and nutritional conditions in early phases of life play a key role in the etiology of these diseases by inducing physiological dysfunctions (Lucas, 1998; Victora et al., 2008; Wells, 2012). Many studies have suggested that external environmental inputs, such as nutrition, may modify the phenotype, leading to physiological adaptations without genetic changes. This phenomenon can be understood in the context of phenotypic plasticity. (West-Eberhard, 2003).

Phenotypic plasticity is molecularly based on epigenetic changes, such as DNA methylation, post-translational histone modifications, and microRNA expression (Wells, 2011). The epigenetic landscape was first described by Conrad Waddington in 1940 who studied the relationship between the cause and effect in genes to produce a phenotype (Jablonka and Lamb, 2002). Currently, this concept is used for the process of gene expression and its link to modifications in the chromatin structure without altering the DNA sequence (Chong and Whitelaw, 2004; Egger et al., 2004). Changes in the chromatin structure are related to the increase or decrease in the electrostatic affinity of the DNA structure. DNA methylation, post-translational modifications of histones, and expression of microRNAs are capable of altering a chromatin structure (Sadakierska-Chudy and Filip, 2015). DNA methylation is related to the addition of methyl groups to DNA cytosine residues, usually cytosine, followed by the guanine residue (CpG dinucleotides), which can produce inhibition of gene expression by impairing transcription factor binding (Waterland and Michels, 2007; Mansego et al., 2013; Chango and Pogribny, 2015; Mitchell et al., 2016). As post-translational histone modifications (acetylation, methylation, ubiquitination, and phosphorylation) correspond to the addition of methyl, acetyl, or other groups to the histone tails, they increase or decrease the electrostatic affinity between histone proteins and DNA, thus promoting a structure of chromatin that is more or less permissive to gene transcription (Bowman and Poirier, 2015). The addition of acetyl groups to histones is regulated by the action of histone acetyltransferases (HATs), while the removal of acetyl groups is catalyzed by histone deacetylases (HDACs) (Graff and Tsai, 2013). MicroRNAs are small endogenous non-coding RNA molecules involved in gene regulation and function in protein-coding introns, non-coding gene introns, or non-coding gene exons; they have been implicated in many cellular processes, including proliferation, apoptosis, differentiation, senescence, and responses to stress and immunological stimuli (D'Ippolito and Iorio, 2013).

This provides the basis for an investigation on how the nutritional aspect can induce these epigenetic changes. The hypothesis is that epigenetic modifications are an extended mechanism that links maternal nutrition to the modulation of phenotypes in the offspring (Mazzio E. A. and Soliman K. F. A., 2014; Szarc vel Szic et al., 2015). Thus, this review will address the main cardiometabolic effects elicited by postnatal hyperlipidic diets in protein-restricted subjects during pregnancy and lactation.

## Maternal Protein Undernutrition and Development of Cardiometabolic Diseases

Developmental origins of health and diseases as proposed by Barker and colleagues in 1986 have been extensively studied as physiological consequences of perinatal nutritional factors (Barker and Osmond, 1986; Barker et al., 1989; Barker et al., 1993; Barker, 2007). This field of research proposes that cardiometabolic diseases can be “programmed” by the “adaptive” effects of both under- and overnutrition during early phases of growth and development, changing the cell physiology in the phenotype but without altering the genotype (Barker et al., 2005; West-Eberhard, 2005; Labayen et al., 2006; Andersen et al., 2009; Biosca et al., 2011). In this context, phenotypic plasticity has molecular basis, epigenetic alterations such as DNA methylation, histone acetylation, and microRNA expression (Wells, 2011). These epigenetic marks are established early in development and can persist for a lengthy period of time (John and Rougeulle, 2018).

Epigenetic modifications are widely hypothesized to be an overarching mechanism linking maternal nutrition to metabolic health phenotypes in the offspring (Mazzio EA. and Soliman KF., 2014; Szarc vel Szic et al., 2015). In this context, a low-protein diet (8% protein) during gestation and lactation has been associated with growth restriction, asymmetric reduction in organ growth, elevated systolic blood pressure, dyslipidemia, and increased fasting plasma insulin concentrations in most studies on rat offspring (Ozanne and Hales, 2004; Costa-Silva et al., 2009; Falcao-Tebas et al., 2012; Leandro et al., 2012; Fidalgo et al., 2013; de Brito Alves et al., 2014; Ferreira et al., 2015; de Brito Alves et al., 2016; Paulino-Silva and Costa-Silva, 2016; Barbosa et al., 2020). However, it is known that the magnitude of the cardiovascular and metabolic outcomes is dependent on both time exposure to a protein restricted-diet (Zohdi et al., 2012; Zohdi et al., 2014) and the growth trajectory throughout the postnatal period (Wells, 2007; Wells, 2011).

A relationship between maternal protein restriction, sympathetic overactivity, and hypertension has been suggested (Johansson et al., 2007; Franco et al., 2008; Barros et al., 2015). Currently, it is well accepted that perinatal protein malnutrition (6–9% protein) raises the risks of hypertension by mechanisms that have been shown to include abnormal vascular function in the adult rat male (Brawley et al., 2003; Franco et al., 2008), altered nephron morphology and function, and stimulation of the renin-angiotensin system in the adult rat male (Nuyt and Alexander, 2009; Siddique et al., 2014) as well as disruption in respiratory control in the rat male at 30 and 90 days of life (Chen et al., 2010; de Brito Alves et al., 2014; Barros et al., 2015; Costa-Silva et al., 2015; Paulino-Silva and Costa-Silva, 2016; de Brito Alves and Costa-Silva, 2018). Offspring from dams subjected to perinatal protein restriction had relevant short-term effects on the carotid body (CB) and sensitivity and respiratory functions as well as enhanced baseline sympathetic activity and amplified ventilatory and sympathetic responses to peripheral chemoreflex activation, prior to the establishment of hypertension (de Brito Alves et al., 2014; de Brito Alves et al., 2015). The mechanism involved in these effects seems to be linked with upregulation of the hypoxic inducible factor (HIF-1α) in the CB peripheral chemoreceptors (Ito et al., 2011; Ito et al., 2012; de Brito Alves et al., 2015; Nogueira et al., 2018). Studies showed that CB peripheral chemoreceptors in malnourished offspring were responsible for the enhanced respiratory frequency and CO_2_ chemosensitivity in early life and the production of autonomic imbalance and the development of hypertension in adulthood (Nogueira et al., 2018). In addition, it was demonstrated that these cardiorespiratory disruptions observed in offspring were attenuated from mothers who performed physical activity during the perinatal period (Nogueira et al., 2019). Regarding epigenetics mechanisms involved in cardiometabolic effects elicited by protein malnutrition, it was described as a decrease in the methylation at various positions of the ACE-1 promoter region in rat brain and an increase in transcription of this gene involved in the renin-angiotensin system and the maintenance of arterial blood pressure (Goyal et al., 2010). Similarly, in humans, the DNA methylation in the ACE gene promoter of peripheral blood leukocytes seems to be significantly lower in children of age ranging from 6 to 12 years born with low birth weight, resulting in greater ACE activity (Ajala et al., 2012; Rangel et al., 2014).

Maternal protein restriction affects insulin sensitivity in the offspring. Previous studies found that in rats, maternal protein restriction throughout pregnancy and lactation induced insulin resistance in the male offspring (Zambrano et al., 2006; Berends et al., 2018). This central insulin resistance is related to reduced protein levels of the p110*β* subunit of phosphoinositide 3-kinase (PI3K) and increased serine phosphorylation of IRS-1 in the arcuate nucleus (ARC) of the hypothalamus. The expression of the gene encoding protein tyrosine phosphatase 1B (PTP1B; Ptpn1) was also increased in the region of the hypothalamus (Berends et al., 2018); the mechanism appeared to increase insulin receptor signaling mediated by protein kinase C (PKC)-*ζ* in skeletal muscle of the offspring of rats fed with a low-protein diet during pregnancy and lactation (Chen et al., 2009). The ability of skeletal muscle to respond to maternal protein restriction is an adaptation to optimize the use of nutrients available during the life-span, and an important response in this process is the activation of genes that ameliorate or compensate for protein deficit by stimulating the expression of glucose transporters and glycolytic and lipolytic enzymes that attenuate the altered function of the mitochondria (da Silva Aragao et al., 2014; Claycombe et al., 2015). At 30 days, the transcriptional key enzymes of the glycolytic pathway were downregulated in extensor digitorum longus muscle in offspring. However, these effects were only observed at 90 days in soleus muscle of rats subjected to protein maternal undernutrition. PDK4 was the enzyme that was more affected. One important finding was that the observed acute (30 days) transcriptional changes did not remain in adult LP rats (90 days), except for PDK4. The robust PDK4 mRNA downregulation, observed in both soleus and EDL, at both ages, and the consequent downregulation of the PDK4 protein expression can be responsible for a state of reduced metabolic flexibility of skeletal muscle in response to maternal low-protein diet (de Brito Alves et al., 2017).

Studies in rodents subjected to perinatal protein malnutrition have observed an impact on liver function with the suppression of gluconeogenesis by a mechanism mediated mainly by the decrease in the level of hepatic phosphoenolpyruvate carboxykinase mRNA (Toyoshima et al., 2010), increase in blood cholesterol and triglycerides in the offspring at 110 days of life, and reduced gene expression for the glucokinase (GCK) enzyme, the glucose sensor in the liver, impairing the detection of glucose levels (Sosa-Larios et al., 2017); these effects have been accompanied by DNA hypomethylation and increased expression of genes involved in lipid metabolism (Radford et al., 2014) and hypomethylation of glucocorticoid receptors and PPAR-*α* promoters, which conditioned changes in the expression of their target genes (Sandovici et al., 2011); at 21 days, the mice showed a reduction in the microRNAs, namely, mmu-miR-615, mmu-miR-124, mmu-miR-376b, and mmu-let-7e, while mmu-miR-708 and mmu-miR-879 were increased after microarray analysis; bioinformatics analysis showed that target genes were mapped to inflammatory pathways, accompanied by elevation of serum levels of tumor necrosis factor-*α* (TNF-α) (Zheng et al., 2017).

## Postnatal Overnutrition and Development of Cardiometabolic Diseases in Maternal Protein-Restricted Subjects

Nutritional transition is a phenomenon well documented in developing countries in the 20th and 21st centuries and has induced a high incidence of the chronic diseases and a high prevalence of obesity (Batista Filho and Rissin, 2003b; Batista Filho and Batista, 2010; Ribeiro et al., 2015; Leocádio et al., 2021). It is evident that protein malnutrition was a health problem in the first half of the 20^th^ century. Now, this has been replaced by a diet enriched in saturated fat or other high-fat diets, predisposing the population to overweight and obesity (Batista et al., 2013). Nowadays, it is suggested that two billion people in the world are overweight and obese individuals, with a major prevalence related to diet-induced obesity, which has been associated with cardiovascular and endocrine dysfunctions (Hotamisligil, 2006; Aubin et al., 2008; Zhang et al., 2012; Ng et al., 2014; Wensveen et al., 2015).

In the late 1980s–1990s, Barker et al. provided epidemiologic evidence of the programming of offspring metabolic syndrome, demonstrating that low birth weight was a significant predictor of adult obesity, diabetes, and cardiovascular disease, for promoting changes in the fetal environment, which can trigger genetic alterations and reflect on the maturation of fetal organs and systems (Barker, 1999; Barker, 2007). In a recent global survey conducted in 30 low-income countries, Abdullah (2015) related that the pattern of body weight gain of the population of developing countries is almost identical to that found in those countries and calculated the estimate of overweight over time, being higher in groups with less and less education. The coexistence of malnourished children and obese mothers in the same residence is a reality in Mexico (Kroker-Lobos et al., 2014), Colombia (Sarmiento et al., 2014), China (Feng et al., 2015), sub-Saharan Africa (Steyn and McHiza, 2014), and also in Brazil (Sousa et al., 2016). Short stature and obesity may reflect malnutrition and a poor quality diet in the first 2 years of life, followed by excess energy intake later in childhood (Abdullah, 2015).

Some studies have tried to mimic the nutritional transition, with peri- and postnatal nutritional mismatch, producing animal models that are based on maternal protein restriction during pregnancy and/or lactation, followed by the consumption of high-fat diets by the offspring, after weaning. Epidemiological studies have demonstrated that low birth weight makes individuals more susceptible to hypercholesterolemia only when combined with postnatal consumption of a high-fat diet (Robinson et al., 2006). These results suggest that the postnatal nutritional environment may affect cholesterol metabolism differently in low birth weight individuals compared to their normal weight peers. One mechanism that may explain the link between prenatal growth and adult disease is a permanent change in gene expression in response to the early environment (Gluckman and Hanson, 2004).

Animal studies have shown that a high-fat diet significantly increased weights and body fat of malnourished rats in gestation and/or lactation, reduced lean body mass, and accentuated plasma leptin, an increase in glucose levels with increased insulin levels and hypertriglyceridemia in male rats (Desai et al., 2015). Assessing the effects of a nutritional transition model with incompatibility between peri- and postnatal diets in mice, Sellayah et al. (2014) offered a control diet (18% protein) or protein-restricted diet (9% protein) during pregnancy and lactation to mothers. After weaning, the male pups began to receive a standard diet (7% fat) or a high-fat diet (45% fat) until 30 weeks of age. The authors observed that offspring maternal protein restriction to those who consumed high-fat diet resulted in an increase in body adiposity, even without changing the total weight or increasing the lipid content in muscle tissue. While the consumption of a high-fat diet by animals that had not suffered previous malnutrition promoted an increase in energy expenditure and expression of proteins related to thermogenesis (uncoupling protein 1 - UCP1; adrenergic receptor beta 3 - β-3AR) in brown adipose tissue, the maternal protein restriction did not show the same response. These results suggested that a mismatch can attenuate diet-induced thermogenesis and contribute to the development of obesity. Animals with maternal protein restriction (5% protein) fed for 4 weeks with a high-fat diet showed a relatively dangerous increase in the white adipose tissue and a decrease in gross gastrocnemius muscle weight in males even without causing changes in bodyweight; in addition, males and females exhibited anxiety-like behaviors (de Almeida Silva et al., 2020).

The increase in white adipose tissue may be one of the factors that contribute to the development of cardiometabolic diseases in maternal protein-restricted individuals. A continuous intake of an HF diet can promote adipocyte hypertrophy and dysfunction and induce the infiltration of pro-inflammatory macrophages in adipose tissue, increasing the production of pro-inflammatory cytokines in this tissue (Ouchi et al., 2011). An increased immune activity is associated with a high consumption of high-fat diets and favors the maintenance of chronic systemic inflammatory processes, originating in adipose tissue (Wensveen et al., 2015; Ertunc and Hotamisligil, 2016; Lyons et al., 2016). Using a mouse model of a prenatal low-protein diet (LP, 8% protein) followed by a normal or postnatal diet high-energy in fat (HE, 45% fat) for 12 weeks, maternal protein restriction added to a high-fat diet interacts to affect growth recovery and leads to an increase of the offspring’s adipocyte tissue, which correlates with the phenotype of inflammation in adipose tissue (Xie et al., 2017). In this study, adipose tissue macrophage infiltration was not affected by the LP diet, as evidenced by the lack of difference in the number of CD68 cells in the adipose tissue. However, after postnatal treatment of HE, there were fewer cells of adipose tissue macrophages M1b subtypes (CD11c + CD206+) in f1 offspring from maternal LP dam than those on the normal protein maternal diet. The maternal LP diet interacts with the postnatal high-fat diet to impact the macrophage phenotypes of the existing adipose tissue, although the prenatal LP diet may not influence the ability of monocyte/macrophage migration from adipose tissue in F1 mouse offspring. At 60 days of age, the interaction between low maternal protein during pregnancy and lactation (9% casein) and offspring’s high-fat diet, after weaning (45% lipids), increased retroperitoneal and epididymal fat deposits and increased the production of nitric oxide by adipocyte macrophages (Alheiros-Lira et al., 2017). Adipose tissue with dysfunctional signaling has been considered to trigger reduced insulin sensitivity (Caricilli et al., 2008; Calder, 2012) and change the plasma and tissue lipid profile (Ralston et al., 2017) in addition to promoting cardiovascular disorders (Wang and Hu, 2017). A high production of nitric oxide has been implicated in the apoptosis of macrophages (Sarih et al., 1993) and pathogenesis of inflammatory diseases (Borges et al., 2013), suggesting high immunoreactivity induced by dietary fatty acids after metabolic programming with maternal protein restriction. Maternal prenatal malnutrition appears to modify the programs of the gene expression profile of offspring adipose tissue over the long term; this, combined with obesogenic nutrition, predisposes prenatal malnourished individuals to an altered lipid metabolism and fat accumulation in adulthood (Lukaszewski et al., 2011).

Maternal protein restriction (8% casein) during pregnancy and lactation followed by a post-weaning HF diet (41% fat) induced an increase in the percentage of visceral fat, reduced insulin sensitivity, and increased food intake in rat males at 90 days of age (Gosby et al., 2010). In an experimental design with Sprague–Dawley rats, Iwasa et al. (2017) demonstrated that the consumption of a post-weaning high-fat diet (62.2% of calories from fat) induced an increase in the serum leptin level and a trend toward a reduction in the hypothalamic POMC mRNA level in the offspring of undernourished dams, whereas it had no effect on the levels of orexigenic factors or other anorexigenic factors. Male mice at 220 days of life malnourished during pregnancy and lactation and fed with a high-fat postnatal diet showed increased gene expression of POMC and MC4R in the hypothalamus and hypomethylation of the POMC promoter in the hypothalamus (Zheng et al., 2021). Vickers et al. (2000) studied the interaction between restricted maternal diet and amplification by a post-weaning high-fat diet and demonstrated that deep adult hyperphagia is a consequence of fetal programming and an essential contributing factor to adult pathophysiology.

At the serum level, the activity of adipokines, such as leptin, in malnourished organisms, presents divergent behavior and suggests action and selective sensitivity, both with endocrine regulation function (do Prado et al., 2009), in both immune (Arroyo Hornero et al., 2020) and physiological systems. A study by Sellayah et al. (2014) sought to evaluate the effects of incompatibility between the fetal nutritional environment poor in proteins and a lipid-rich environment in the post-weaning period; Wistar rats received a control diet (18% casein) or with protein restriction (9%) during pregnancy and standard diet during lactation. Pups, including males and females, started to receive a control diet (7% lipids and 15% casein) or a high-fat diet (45% lipids and 26% casein), from weaning to 16 weeks of age, when they were evaluated. The study demonstrated that the nutritional mismatch exacerbates the increase in blood pressure promoted by maternal malnutrition, regardless of increased body adiposity, glucose, or leptin levels, modulated upward only by the high-fat diet and not by the maternal diet during pregnancy. Post-weaning hyperlipidic diets have numerous impacts on glycolipid metabolism in offspring with maternal protein restriction, insulin intolerance, decreased insulin sensitivity, higher triglyceride/high-density lipoprotein ratio and high levels of leptin and interleukin-6 in adipose tissue and low adiponectin (Wu et al., 2020).

Another organ that appears to be particularly affected by the nutritional transition and which is at the central core of the development of a range of metabolic diseases is the liver. The liver is sensitive to dietary modulations. A shortage of amino acids, for example, has been related to liver diseases (Nishi et al., 2018). In an experimental model of protein malnutrition, hepatocytes isolated from rats fed with 5% casein protein for 14 days showed increased triglyceride synthesis (Taguchi et al., 2017). Transcription factors, such as the carbohydrate-sensitive response element-binding protein (ChREBp), peroxisome proliferator-activated receptors (PPAR), and the sterol regulating element-binding protein, (SREBp) respond to excessive lipid consumption, and they control energetic homeostasis and can activate pathways related to lipogenesis and inflammatory processes in liver tissue (Mello et al., 2016; Shimano and Sato, 2017; Li et al., 2019). Chmurzynska et al. (2012) showed that the altered response to a high-fat diet programmed by maternal nutrition during pregnancy was detected as an altered gene expression in 10-week-old rats and central adiposity in 16-week-old rats. Hepatic transcription of PPARγ in response to the high-fat diet was dependent on maternal nutrition. Male rats subjected to maternal protein restriction and fed with a high-fat diet showed an increase in PPAR levels, while rats with a normoprotein maternal diet and a postnatal diet rich in fatty acids showed a reduction in PPAR levels. Likewise, activation of the *PPARα* gene by a high-fat diet was dependent on prenatal nutrition (Chmurzynska et al., 2012).

Assessing the structure of liver tissue, a study by Souza-Mello et al. (2007) demonstrated that nutritional mismatch with a low protein maternal diet (9% during pregnancy and lactation) and a postnatal diet rich in lipids (50% of the total caloric component) induces an increase in blood pressure and gonadal adiposity, in addition to worsening liver steatosis in male rats. However, the mechanisms are still unclear, but they may be related to an increased activity of lipid synthesis and oxidation as a strategy to save proteins in conditions of scarcity and in the regulation of transcription factors and gene expression related to glycolipid metabolism. In an experimental study with sheep, a 50% reduction in dietary proteins during pregnancy and lactation, followed by post-weaning consumption of a high-fat diet (38% fat), increased the hepatic triglyceride content in lambs, with permanent reduction induction in the expression of genes that regulate energy homeostasis (*GLUT1*, *PPAR*, *SREBP1c*, and phosphoenolpyruvate carboxykinase gene—*PEPCK*) (Souza-Mello et al., 2007).

During fasting states, the use of fatty acids as an energy substrate occurs through *β*-oxidation, mainly in a mitochondrial environment (Reddy and Rao, 2006), with the action of key enzymes such as acyl-CoA dehydrogenase, enoyl-CoA hydrolase, and *β*-hydroxycil CoA-dehydrogenase (Jones, 2016). Elevated insulin and glucose levels regulate the synthesis of triacylglycerols, stimulating the activity of transcription factors such as ChREBp and the sterol regulatory element-binding protein SREBp and activated by peroxisome proliferator receptors PPAR. These factors increase the expression of lipogenic enzymes such as acetyl-coenzyme A carboxylase (Zhao et al., 2012) and fatty acid synthase FAS (Canbay et al., 2007). Imbalances in transport mechanisms or in the activation of transcription factors can then induce excessive accumulation of triglycerides within hepatocytes and trigger liver diseases (Titchenell et al., 2017).

**FIGURE 2:**
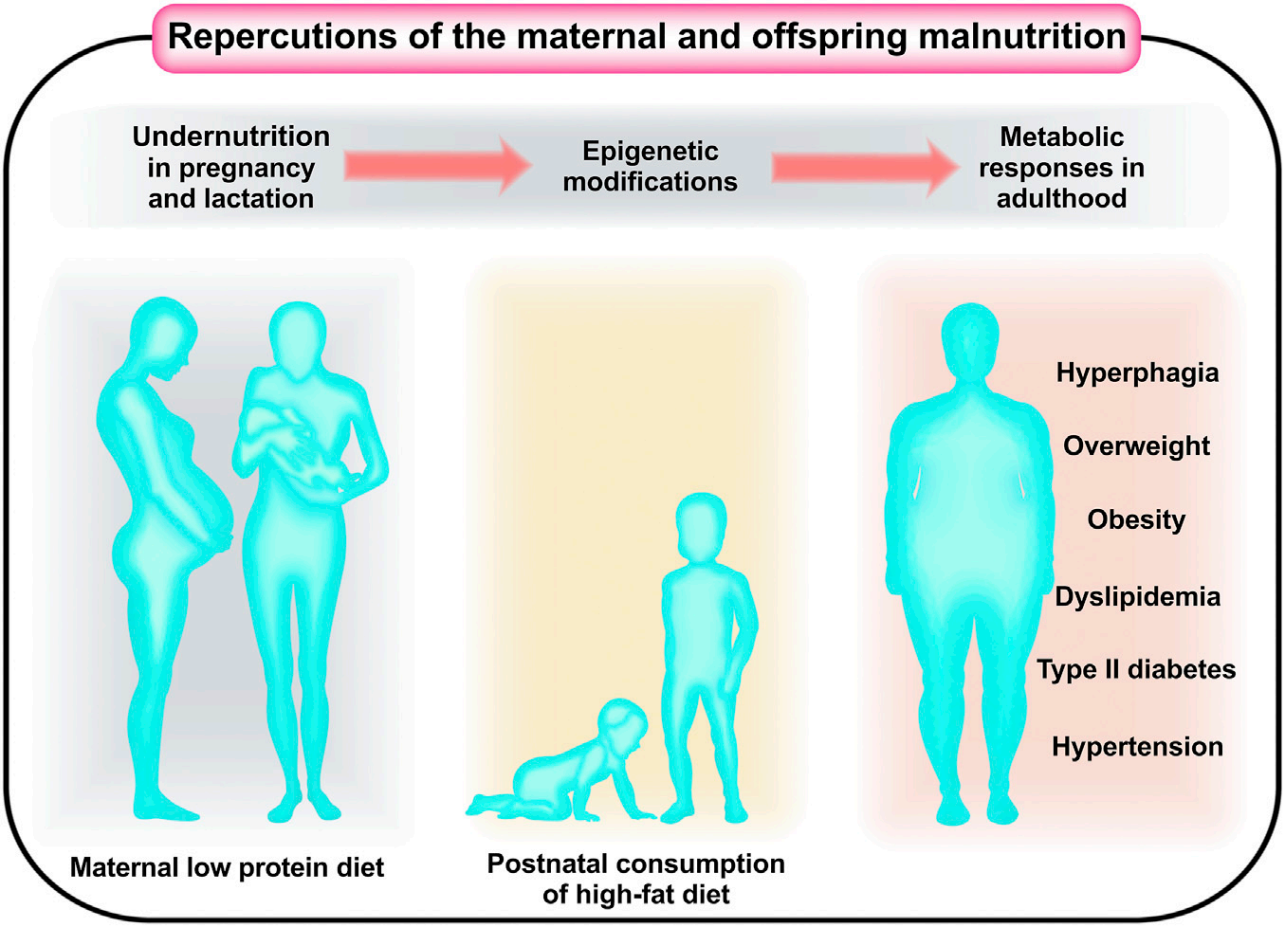
Graphic summary of the repercussions of the postnatal consumption of high-fat diets in individuals subjected to protein restriction during pregnancy and lactation.

The increase in energy levels induces a reduction in mitochondrial oxidative activity, while the increase in lipid peroxidation induces damage to mitochondria. In the liver, a maternal protein restriction of Wistar rats (casein 8%) during pregnancy and lactation, followed by a diet with post-weaning HF until 90 days of life (32% of the caloric percentage originated in lipids and 59% at more than saturated fatty acids), induced a reduction in all respiratory states of liver mitochondria, (Vickers et al., 2000) greater mitochondrial edema compared to controls enhanced after the addition of Ca2^+^ and prevented in the presence of EGTA (calcium chelator) and cyclosporine A (transition pore inhibitor of mitochondrial permeability), and greater oxidation of liver proteins and lipid peroxidation, with a reduction in catalase and glutathione peroxidase activities. These data suggest that adult rats subjected to maternal protein restriction were more susceptible to liver mitochondrial damage caused by a diet rich in saturated fatty acids after weaning (Simoes-Alves et al., 2019). Figure 2 summarizes the repercussions of the consumption of high-fat diets postnatally in individuals subjected to protein restriction during pregnancy and lactation.

## Conclusion

A shift in the nutritional status of the perinatal and postnatal environment induces accelerated recovery growth and adjustments in autonomic modulation and insulin sensitivity as well as mitochondrial dysfunction. A post-weaning high-caloric/high-fat diet potentiates these adjustments, exacerbating deleterious changes in important metabolic organs, namely, hepatic, adipose, and muscular tissue. The epigenetic repercussions of postnatal metabolic overload may be etiological sources of cardiometabolic diseases, which affect the subjects suffering nutritional transition.
